# The Use of Aquacel® Ag for the Management of Partial-Thickness Burns in a Tertiary Center

**DOI:** 10.7759/cureus.105049

**Published:** 2026-03-11

**Authors:** Zain Bukamal, Taraf Jaro

**Affiliations:** 1 Plastic and Reconstructive Surgery, Salmaniya Medical Complex, Manama, BHR; 2 Plastic and Reconstructive Surgery, Royal College of Surgeons in Ireland – Medical University of Bahrain, Manama, BHR

**Keywords:** antimicrobial dressing, burns and plastic surgery, silver hydrofiber, silver sulfadiazine dressing, superficial partial-thickness burn

## Abstract

Background: Silver-based dressings provide a broad range of antimicrobial and re-epithelialization benefits, making them beneficial for the rapid regeneration of wounds. Their benefits have been observed for the management of burns, in particular Aquacel® Ag, and they display antimicrobial, cosmetic, and cost-effective enhancements in the clinical setting. These characteristics provide great potential for Aquacel® Ag use in hospitals with high patient loads, such as Salmaniya Medical Complex in Bahrain.

Objective: This study investigates the efficacy of Aquacel® Ag relative to 1% silver sulfadiazine for the management of partial-thickness burns across multiple parameters, with the goal of encouraging the establishment and implementation of guidelines that incorporate Aquacel® Ag as a key intervention in the management of partial-thickness burns.

Design and methods: This retrospective cohort study gathered data on various demographic and burn-progression parameters, including patients’ age, total body surface area, days of hospital admission, and burn swab culture. The study has been carried out at the Salmaniya Medical Complex, Bahrain. The primary outcomes assessed were infection rates and the number of days between dressing changes.

Results: The mean duration of inpatient admission was significantly longer in the silver sulfadiazine-treated group, 12.43 ± 11.291 days, than in the Aquacel® Ag group at 4.97 ± 5.195 days (p<0.001). Additionally, the rates of positive burn swabs were significantly higher in the 1% silver sulfadiazine group than the Aquacel® Ag group, at 64.3% and 7.8%, respectively (p<0.0001).

Conclusion: These findings encourage the establishment and implementation of guidelines incorporating Aquacel® Ag as a key intervention in the management of partial-thickness burns in inpatient and outpatient settings.

## Introduction

Wound healing requires a sterile, dry environment to ensure maximum efficacy of the physiological process. Desired outcomes include functionality, cosmetic appearance, and a non-infectious status. In outpatient and emergency department settings, an additional factor in wound healing is patient satisfaction via decreased pain and shorter wound-healing time. Burns, in particular, occur when skin comes into contact with heat sources, including scalding liquids, flames, electricity, and chemical substances [1,2]. However, additional factors such as the duration of contact, the total body surface area (TBSA) affected, and temperature affect the characteristics of burn wounds. Often, longer durations of contact over a greater surface area increase the morbidity and mortality of the wound [3,4]. The plethora of risk factors increases the susceptibility of children and the elderly alike, demanding a rapid, accessible solution for effective management of burns.

Over the last decade, the use of silver in dressings has become an effective option to achieve this goal. With a wide range of indications for their use, including the management of burns, surgical wounds, diabetic foot, and pressure ulcers, their prominence has gained traction in recent years. Silver-based dressings are topical wound dressings available as foam dressings, hydrocolloids, barrier layers, and charcoal cloths. The silver in the dressing is activated by the presence of exudate and bacteria in the wound, the extent of which determines how much silver is released into the wound [5,6]. Their antimicrobial properties have been shown, especially through significant reduction in surgical site infections and granuloma and exudate formation while enhancing epithelization of superficial partial-thickness burns [7,8]. Silver-based dressings have contributed to enhanced healing outcomes in burn management, which makes them an ideal candidate for use in emergency departments and in burn units.

Aquacel® Ag is a widely used silver dressing that removes excess exudate and provides an effective pathogenic barrier to reduce pathogenic load. Its hydrofiber technology is a fibrous composition of sodium carboxymethylcellulose (CMC) with 1.2% ionic silver that creates a cohesive gel upon contact with exudative wounds, providing its hydrocolloid property [9]. This hydrofiber structure is completed with a layer of polyurethane, a waterproof substance, ensuring an environment with optimal conditions for effective wound healing [10]. 

Salmaniya Medical Complex (SMC) is a central provider of care for a significant portion of Bahrain’s population. Many patients rely on SMC for both emergency needs and tertiary care. While Aquacel® Ag is available and used for the treatment of partial-thickness burns in the burns unit and outpatient clinic, there are no guidelines in place to standardize its use. In 2011, a guideline consensus was compiled, comprising management guidelines from Europe, North America, the Far East, South Africa, and Australia, to provide internationally recognized guidance on the appropriate use of silver dressings, based on clinical experience and all available published evidence [11].

Through this study, we aim to display the efficacy of Aquacel® Ag relative to alternative dressings, with the goal to encourage the establishment and implement guidelines incorporating Aquacel® Ag as a key intervention in the management of partial-thickness burns.

## Materials and methods

Study design

This study was conducted as a retrospective cohort study using convenience sampling. Patient data were obtained from the I-SEHA electronic medical record system at SMC, Bahrain. The study evaluated outcomes in patients admitted to the burns unit between January 1, 2022, and December 31, 2022, who were treated with either Aquacel® Ag or silver sulfadiazine dressings.

Study setting

SMC is a tertiary care government hospital in Bahrain with a dedicated burns unit that manages acute burn injuries. Both Aquacel® Ag and silver sulfadiazine are routinely used in the unit as part of standard burn care.

Study population and sample size

The study population included all patients admitted to the burns unit during the study period who met the inclusion criteria. A total of 78 patients were included; 64 were treated with Aquacel® Ag and 14 with 1% silver sulfadiazine. All the included patients had partial-thickness burns. 

Inclusion criteria

Patients were included if they: presented with partial-thickness burns, had a TBSA involvement between 2% and 25%, were admitted to the burns unit between January and December 2022, and were treated exclusively with either Aquacel® Ag or silver sulfadiazine dressings during their admission. Silver sulfadiazine was selected as the comparator due to its frequent and longstanding use as a standard topical burn dressing in the burns unit.

Exclusion criteria

To minimize confounding factors affecting wound healing, patients were excluded if they had concomitant traumatic injuries; had comorbidities known to impair wound healing, including diabetes mellitus, malignancy, or immunocompromised states; sustained chemical or electrical burns; or required surgical intervention for burn management, such as excision or skin grafting. These exclusions were applied to ensure comparability between treatment groups and to focus on conservatively managed partial-thickness burns. To rule out external factors affecting the rate of wound healing, exclusion criteria include concomitant trauma, comorbidities (diabetes mellitus, malignancies, or being immunocompromised), having sustained chemical or electrical burns, or having required surgical treatment of the wound [12].

Study measures and outcomes

The primary outcome measure was the number of days between dressing changes, used as a surrogate marker for the dressing’s ability to contain wound exudate and maintain an optimal healing environment. Secondary outcomes include rates of infection, organisms cultured from burn swabs, and duration of hospital admission.

All outcomes were extracted from clinical notes, nursing documentation, and microbiology reports within the I-SEHA system.

Data collection

Data collected included patient demographics, burn characteristics (TBSA and burn depth), type of dressing used, frequency of dressing changes, microbiology results, and length of hospital stay. Data were anonymized before analysis to maintain patient confidentiality.

Statistical analysis

Statistical analysis was performed using appropriate statistical software. Continuous variables are presented as means ± standard deviations, and categorical variables as frequencies and percentages.

Between-group comparisons were performed using independent samples t-tests for continuous variables (age, length of hospital stay, and mean days between dressing changes) and chi-square tests for categorical variables (sex distribution and incidence of infection). All tests were two-tailed, and a p-value of <0.05 was considered statistically significant.

H0: No significant difference has been reported in the number of days between dressing changes when using Aquacel® Ag or silver sulfadiazine for the management of partial-thickness burns.

H1: The use of Aquacel® Ag results in a significantly greater number of days between dressing changes for the management of partial-thickness burns due to its increased ability to contain exudate within the wound.

Dressing application

After irrigating the burn wound with normal saline and drying it adequately, the Aquacel® Ag sheet is applied to the wound bed. Silver sulfadiazine cream is similarly applied to the area of burn directly. Dry dressing gauze is then applied over the used agent, followed by a final layer of crepe bandage. 

Ethics statement

Ethical approval was obtained from the Research Committee for Government Hospitals in Bahrain before study commencement. 

## Results

Seventy-eight patients were included in this retrospective study, in which 64 patients were treated with Aquacel® Ag and 14 with silver sulfadiazine. Fifty-three patients were male, while 25 were female. Regarding patient demographics, the Aquacel® Ag group was younger than the silver sulfadiazine group (Table 1). Additionally, both groups were more greatly comprised by males. The mean TBSA on admission was higher in the silver sulfadiazine group (11.36 ± 10.112%) than in the Aquacel® Ag group (6.64 ± 4.858%). Amounting to a total of 56.4% of cases, the most common wounds were scald burns (Table 2). The most frequently-occurring sites included unilateral upper and lower limbs (n=25), face and/or neck (n=22), and back (n=25) (Table 2). This interesting observation is likely owed to the mechanism of injury of accidentally spilling hot liquids onto children. On the other hand, the least occurring site for burn in this population is the perineum (n=6). Mean duration of admission was significantly longer for silver sulfadiazine-treated patients at 12.43 ± 11.291 days than for Aquacel® Ag at 4.97 ± 5.195 days (p<0.001).

**Table 1:** Patient demographics Ag, silver.

Demographics			Aquacel® Ag	Silver sulfadiazine	Total
Mean age (years)			19.03 ± 18.96	30.71 ± 15.11	-
Gender	Male	Frequency	45	8	53
		Percentage	70.3%	57.1%	67.9%
	Female	Frequency	19	6	25
		Percentage	29.7%	42.9%	32.1%
-	Total	Frequency	64	14	78

**Table 2:** Burn classifications and sites Ag, silver.

Burn classification and site			Aquacel® Ag	Silver sulfadiazine	Total
Burn classification	Scald	Frequency	38	6	44
		Percentage	59.4%	42.9%	56.4%
	Flame	Frequency	22	7	29
		Percentage	34.4%	50.0%	37.2%
	Contact	Frequency	2	1	3
		Percentage	3.1%	7.1%	3.8%
	Friction	Frequency	2	0	2
		Percentage	3.1%	0.0%	2.6%
Burn site	Unilateral upper limb		25	0	25
	Bilateral upper limbs		14	4	18
	Unilateral lower limb		18	7	25
	Bilateral lower limbs		6	3	9
	Face/neck		21	1	22
	Posterior thorax		24	1	25
	Anterior thorax		10	2	12
	Abdomen		5	3	8
	Perineum		5	1	6

Differences in age between groups were assessed using an independent samples t-test (t = −2.49), sex distribution was compared using a chi-square test (χ²=0.91), and length of hospital stay was analyzed using an independent samples t-test (t = −2.42, p<0.001).

Additionally, the occurrence of infection was significantly greater in the 1% silver sulfadiazine group than in the Aquacel® Ag group, at rates of 64.3% and 7.8%, respectively (Table 3). Swab cultures of the burn wounds further showed that six cases treated with 1% silver sulfadiazine yielded mixed growth cultures, while only one case treated with Aquacel® Ag was positive for mixed growth, despite more data collected for patient use of the latter (Table 3). Being the most frequently observed isolated organism colonizing burns in the burns unit, *Staphylococcus aureus* infections comprised 7.7% of infections. Of them, 2.6% were methicillin-resistant *S. aureus* infections, while the remaining 5.1% were non-resistant variations of the microbe (Table 3). Significance of the results at p<0.0001 was supported by Chi-square analysis.

**Table 3:** Rates of infection and burn swab cultures MRSA, methicillin-resistant *Staphylococcus aureus.*

Incidence of infection and burn swab culture	Result	Category	Aquacel® Ag	Silver sulfadiazine	Total
Incidence of infection	Positive	Frequency	59	5	64
		Percentage	92.2%	35.7%	82.1%
	Negative	Frequency	5	9	14
		Percentage	34.4%	50.0%	37.2%
Burn swab culture	Sterile	Frequency	60	5	65
		Percentage	93.8%	35.7%	83.3%
	Non-resistant *Staphylococcus aureus*	Frequency	2	2	4
		Percentage	3.1%	14.3%	5.1%
	Mixed growth	Frequency	1	6	7
		Percentage	1.6%	42.9%	9.0%
	MRSA	Frequency	1	1	2
		Percentage	1.6%	7.1%	2.6%

**Figure 1:**
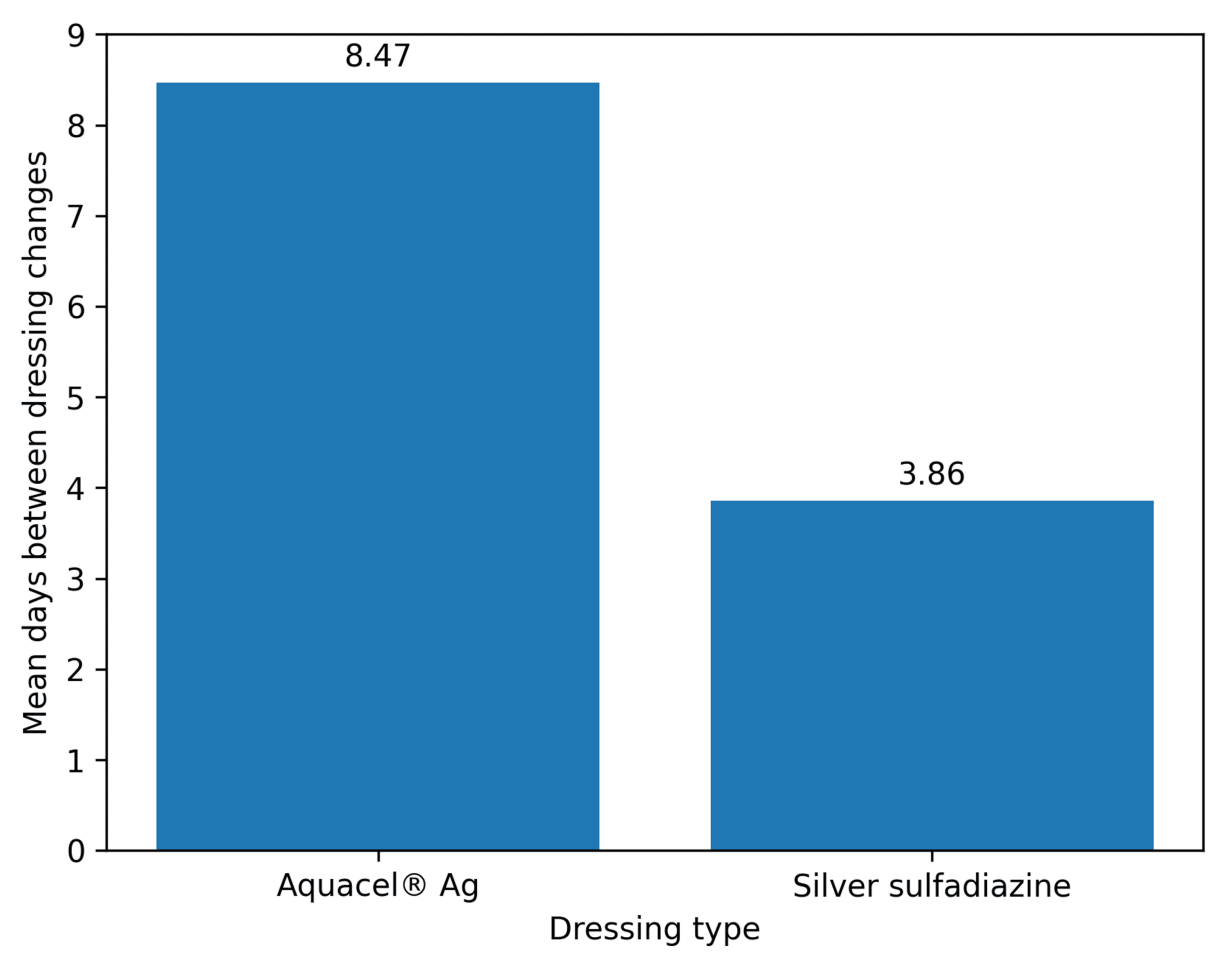
Mean days between dressing changes of Aquacel® Ag and silver sulfadiazine dressings The mean interval between dressing changes was 8.47 days for Aquacel® Ag dressings, compared with 3.86 days for silver sulfadiazine (Flamazine®) dressings.

A significant difference in the mean number of days between dressing changes was observed between patients using Aquacel® Ag (8.47 ± 4.00) and those using 1% silver sulfadiazine (3.86 ± 3.920), with p<0.001 (Figure 1). Given this result, we reject H0 and accept the alternative hypothesis that Aquacel® Ag results in a significantly longer duration between dressing changes, alluding to its increased wound healing efficacy.

## Discussion

Multiple studies have described the efficacy of Aquacel® Ag compared with various alternative dressings, including standard paraffin gauze dressings, silver sulfadiazine, Acticoat™ (an alternative silver-based dressing), and even homeopathic alternatives such as honey [9-12]. Lau et al. compared Aquacel® Ag with standard paraffin gauze dressings in the healing of pediatric partial-thickness burns [12]. Results included significantly shorter mean hospital stay durations, at 14.26 ± 1.90 days for the Aquacel® Ag group, compared with 23.45 ± 3.26 days for the standard paraffin gauze dressing group. Mean frequency of dressing changes further favored the Aquacel® Ag group, with 5.67 ± 0.77 Aquacel® Ag dressing changes compared with 20.59 ± 2.93 paraffin gauze dressing changes (p=0.002). In line with the findings in our investigation, these observations allude to the efficacy of Aquacel® Ag in patients, regardless of demographic differences. Such results have been consistently corroborated by trials investigating the effects of Aquacel® Ag on burns in outpatient and inpatient settings, as well as post-operatively following arthroplasties, breast malignancy wounds, and colorectal procedures. The measured parameters for efficacy include the day of wound closure, pain scores at each dressing change, costs of hospital stay and patient transportation, and infection of the wound [13]. 

The literature almost unanimously favors Aquacel® Ag over a plethora of alternative interventions, owing to the composition of the Aquacel® Ag dressing. Gel formation by sodium CMC upon exudative contact is coupled with its simultaneous ability to absorb the exudate. The waterproof barrier provided by polyurethane externally encapsulates the wound [14]. In doing so, the wound and its adjacent structures such as delicate regenerated tissue are protected from exudative destruction. These properties lend themselves to the benefit of painless dressing removals of Aquacel® Ag and faster epithelialization rates relative to gauze dressings [15]. Additionally, the choice to impregnate the dressing with ionic silver, Ag+, further provides an antimicrobial property. In its cationic state, silver binds to bacterial cell walls and inhibits cytochrome transport needed for respiration, preventing aerobic bacterial growth [16]. However, Ag+ further binds to bacterial DNA, inhibiting replication and providing protective benefits against aerobic, gram-positive, gram-negative, and anaerobic bacteria [17]. Aquacel® Ag has been described as a broad-spectrum antibiotic without the negative implication of microbial drug resistance [17]. 

In future replications, a prospective cohort design would provide the opportunity for qualitative observations of wounds throughout admission and follow-up to monitor for hypertrophic scar formation, granulomas, and epithelization. Observing longitudinal progression allows observation of a more standardized endpoint, strengthening internal validity. The endpoint for most clinical trials assessing dressing efficacy is complete healing, as determined by the US Food and Drug Administration or FDA. Additionally, omitting data collected on patients with comorbidities, especially metabolic disease, decreased its external validity - hence why the mean age was <30 years despite greater variability in our national distribution. Expansion to other hospitals nationally would further improve the generalizability of the findings.

Limitations of our study include a gap in population size between the two patient groups. Also, photographs of the burns before and after treatment with Aquacel® Ag and silver sulfadiazine would have been helpful in displaying the final healed burn wounds. Another limitation was that patients with comorbidities were excluded, which further reduced the population size. In future studies in our center, patients with comorbidities in concordance with burns need to be further assessed and quantified in order to analyze the impact of burns on those with common and/or chronic comorbidities. 

## Conclusions

We have observed in our burns unit that Aquacel® Ag has significantly shortened hospital stays, a lower incidence of burn wound infection, and a reduced mean number of days between dressing changes (p<0.001) compared to silver sulfadiazine dressing. Advantages of Aquacel® Ag use include its broad range of antimicrobial activity, thus reducing or preventing infection. The amount of wound exudate influences the release of silver into the wound. Through its hydrocolloid structure, the dressing absorbs exudate and provides a sustained release of silver.

Despite Aquacel® Ag having a higher cost per unit size than other dressings, the shorter length of hospital stays and fewer dressing changes result in a net cost advantage compared to other dressings. For poorly resourced hospitals, low- and middle-income countries, or hospitals central to communities, this further reiterates their efficiency. In our center, the high patient load means available beds and knowing the necessary resources are crucial to maintain its dependability; therefore, increased management with Aquacel® Ag would improve both inpatient and outpatient outcomes. Future implications of this study include the establishment and implementation of guidelines outlining streamlined Aquacel® Ag use for burn patients in SMC and nationally.
